# Data of oxygen- and pH-dependent oxidation of resveratrol

**DOI:** 10.1016/j.dib.2016.09.012

**Published:** 2016-09-15

**Authors:** Annabell Plauth, Anne Geikowski, Susanne Cichon, Sylvia J. Wowro, Linda Liedgens, Morten Rousseau, Christopher Weidner, Luise Fuhr, Magdalena Kliem, Gail Jenkins, Silvina Lotito, Linda J. Wainwright, Sascha Sauer

**Affiliations:** a Otto Warburg Laboratory, Max Planck Institute for Molecular Genetics, 14195 Berlin, Germany; b Unilever R&D, Colworth Science Park, Sharnbrook, Bedfordshire MK44 1LQ, UK; c University of Würzburg, CU Systems Medicine, Josef-Schneider-Straße 2, Building D15, 97080 Würzburg, Germany; d Laboratory of Functional Genomics, Nutrigenomics and Systems Biology, BIMSB and BIH Genomics Platforms, Max-Delbrück-Center for Molecular Medicine, Robert-Rössle-Straße 10, 13125 Berlin, Germany

## Abstract

We show here if under physiologically relevant conditions resveratrol (RSV) remains stable or not. We further show under which circumstances various oxidation products of RSV such as ROS can be produced. For example, in addition to the widely known effect of bicarbonate ions, high pH values promote the decay of RSV. Moreover, we analyse the impact of reduction of the oxygen partial pressure on the pH-dependent oxidation of RSV. For further interpretation and discussion of these focused data in a broader context we refer to the article “Hormetic shifting of redox environment by pro-oxidative resveratrol protects cells against stress” (Plauth et al., in press) [1].

**Specifications Table**

**Table:** 

Subject area	*Chemistry, Biology*
More specific subject area	*Redox Chemistry, Cell Biology, Biochemistry*
Type of data	*Figure*
How data was acquired	*Plate reader (POLARstar Omega by BMG LABTECH),* CO_2_ Incubator Model CB 60 (Binder)
Data format	*Analysed*
Experimental factors	50 µM *RSV was incubated in water with/without NaHCO*_*3*_*at various pH and oxygen levels.*
Experimental features	*Optical density was measured at different time points at characteristic wavelengths* (308 nm, 390 nm, 420 nm)*.*
Data source location	*Berlin, Germany*
Data accessibility	*Data is within this article and can be found at bioRxiv (*http://dx.doi.org/10.1101/045567, http://biorxiv.org/content/early/2016/03/24/045567*)*

**Value of the data**

• Time- and pH-dependent oxidation data of RSV can be used to assess physiologically relevant effects.
• The influence of oxygen partial pressure on the oxidation of RSV can be assessed for physiological context.
• Pro-oxidative features of RSV shall be tested prior interpretation of physiological effects of RSV.

## 1 Data

**Fig. 1:**
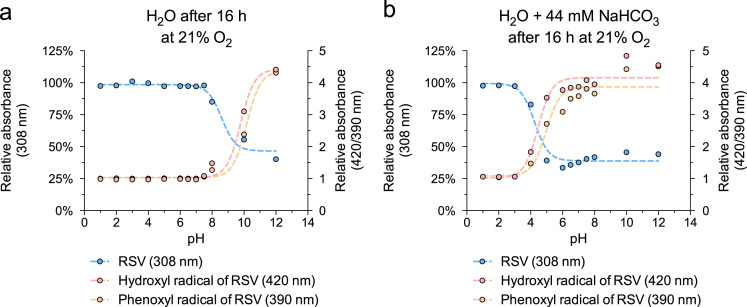
Oxidation of RSV is highly pH-dependent at 37 °C with 21% oxygen. RSV (50 μM) incubated for 16 h in H_2_O without (a) or with 44 mM NaHCO_3_ (b) at divers pH levels at 37 °C. Amounts of RSV and suggested reaction products (hydroxyl radical: 420 nm; phenoxyl radical: 390 nm) detected at characteristic absorbance maxima (Li et al. [2]). pH values were adjusted using HCl and NaOH. Values are mean (*n*=3).

**Fig. 2:**
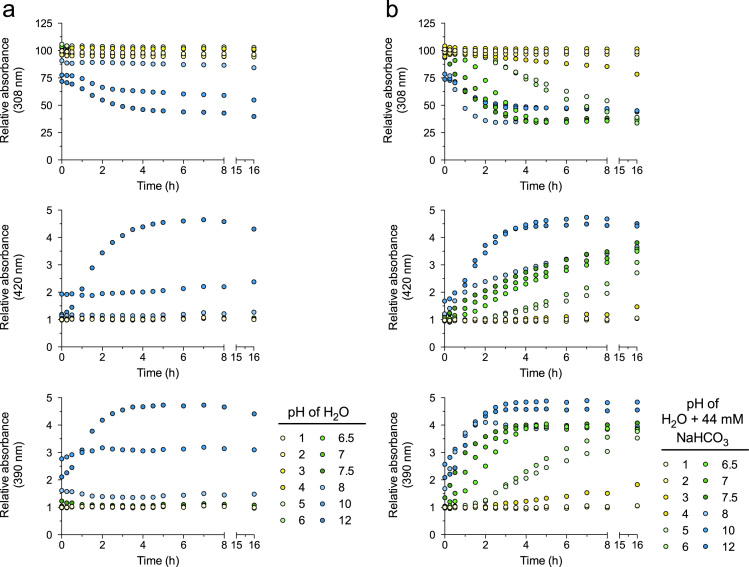
Kinetic oxidation of RSV. RSV (50 μM) was incubated for 16 h in H_2_O without (a) or with 44 mM NaHCO_3_ (b) at divers pH levels at 37 °C with 21% oxygen. Amounts of RSV and suggested reaction products (hydroxyl radical: 420 nm; phenoxyl radical: 390 nm) detected at characteristic absorbance maxima. pH values were adjusted using HCl and NaOH. Values are mean (*n*=3).

**Fig. 3:**
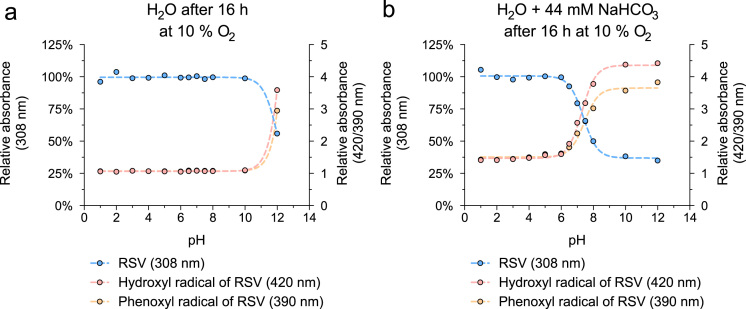
Oxidation of RSV at 37 °C with 10% oxygen. RSV (50 μM) was incubated for 16 h in H_2_O without (a) or with 44 mM NaHCO_3_ (b) at divers pH levels at 37 °C. Amounts of RSV and suggested reaction products (hydroxyl radical: 420 nm; phenoxyl radical: 390 nm) detected at characteristic absorbance maxima. pH values were adjusted using HCl and NaOH. Values are mean (*n*=3).

**Fig. 4:**
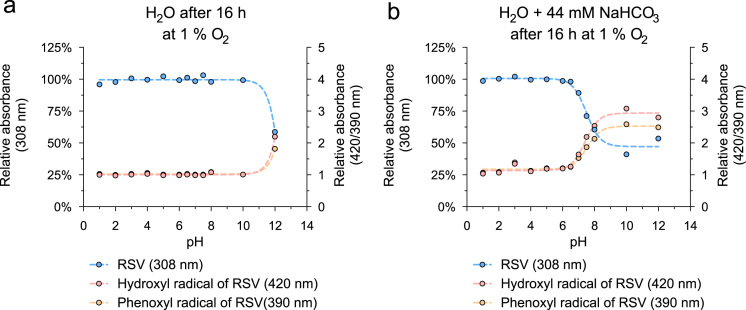
Oxidation of RSV at 37 °C with 1% oxygen. RSV (50 μM) incubated for 16 h in H_2_O without (a) or with 44 mM NaHCO3 (b) at divers pH levels at 37 °C. Amounts of RSV and suggested reaction products (hydroxyl radical: 420 nm; phenoxyl radical: 390 nm) detected at characteristic absorbance maxima. pH values were adjusted using HCl and NaOH. Values are mean (*n*=3).

Here, we analyzed pro-oxidative properties of RSV that can lead to cell-protection via concerted defense mechanisms (1). RSV (50 µM) was incubated for indicated time periods without or with 44 mM NaHCO_3_ at various pH and oxygen partial pressures. Absorbance of RSV and its oxidation products was measured at characteristic maxima: RSV 308 nm, hydroxyl radical of RSV 420 nm, phenoxyl radical of RSV 390 nm. The oxidation of RSV after 16 h at 37 °C with 21% oxygen was highly pH-dependent (Fig. 1) and was accelerated in the presence of 44 mM NaHCO_3_ (Fig. 1b). A detailed kinetic analysis of the pH-dependent oxidation of RSV and the generation of oxidation products is shown in Fig. 2. In addition, the oxidation of RSV was measured at reduced oxygen partial pressure (at 10% oxygen see Fig. 3, at 1% oxygen see Fig. 4).

## 2 Experimental design, materials and methods

### 2.1 Materials

3,5,4׳-trihydroxy-trans-stilbene (resveratrol, RSV) was purchased from Cayman Chemical (Biomol, Hamburg, Germany).

### 2.2 pH-dependent oxidation of resveratrol (cell-free)

The time-dependent oxidation of 50 µM RSV in ddH_2_O with or without 44 mM sodium bicarbonate (NaHCO_3_) was analysed using the POLARstar Omega (BMG LABTECH) at 37 °C. Samples were transferred (150 µl/well) into a UV-Star 96-well plate (# 655801, Greiner Bio-one) for kinetic and spectral measurement (between 230 and 550 nm, Δ*λ* 2 nm). The pH of each solution was adjusted from 1 to 12 using HCl and NaOH. In accordance to Li et al. [2] oxidation products of RSV, a short-lived hydroxyl radical adduct of RSV (characteristic absorbance maximum: 420 nm) and the relatively stable 4׳-phenoxyoxyl radical (characteristic absorbance maximum: 390 nm), were monitored. For data analyses in GraphPad Prism 5.0 signals were background-subtracted and normalised to vehicle control. Data were fitted (dashed line) using GraphPad Prism 5.0 with a Hill slope of −1 according to equation:

$Y=\mathrm{Bottom}+\frac{\left(\mathrm{Top}-\mathrm{Bottom}\right)}{\left(1+10^{\left(X-\log{\mathrm{IC}}_{50}\right)}\right)}$

### 2.3 Oxygen partial pressure-dependent oxidation of resveratrol (cell-free)

96-well plates prepared for the determination of the pH-dependent oxidation of resveratrol (see pH-dependent oxidation of RSV) were incubated at 37 °C at atmospheric oxygen levels (~ 21% O_2_), slightly reduced oxygen partial pressure (10% O_2_, mimicking conditions in the blood vessels), or highly reduced oxygen levels (1% O_2_, resembling tissue or tumour microenvironment). For experiments with reduced oxygen partial pressure, plates were incubated at corresponding oxygen levels using a CO_2_ Incubator Model CB 60 (Binder, Tuttlingen, Germany). For spectral measurements plates were quickly analysed (<2 min) using the POLARstar Omega (BMG LABTECH) at 37 °C. Afterwards the plates were further incubated at indicated conditions. In accordance to Li et al. [2] oxidation of RSV and subsequent reaction products were monitored. For data analyses in GraphPad Prism 5.0 signals were background-subtracted and normalised to vehicle control. Data were fitted (dashed line) using GraphPad Prism 5.0 with Hill slope=−1 according to equation:

$Y=\mathrm{Bottom}+\frac{\left(\mathrm{Top}-\mathrm{Bottom}\right)}{\left(1+10^{\left(X-\log{\mathrm{IC}}_{50}\right)}\right)}$
