# Supplementary material for: Data of oxygen- and pH-dependent oxidation of resveratrol
Source: Data Brief. 2016 Sep 15;9:433–7. doi: 10.1016/j.dib.2016.09.012 (PMC5037118; doi:10.1016/j.dib.2016.09.012)
Supplement: Supplementary file 1 — Supplementary material [file mmc1.docx]

**Declaration of interest**

This work was supported by the German Ministry for Education and Research (BMBF, grant number 0315082, 01EA1303) the National Genome Research Net (NGFN, grant number 01 GS 0828), the European Union [FP7, under grant agreement n° 262055 (ESGI)], and Unilever R&D. None of the funders has a financial interest. Drs. Jenkins, Lotito and Wainwright are employees of Unilever, a company that amongst other develops and sells dermatological and nutritional products.
